# Interlaboratory comparison of a multiplex bead assay against the *Chlamydia trachomatis* antigen Pgp3 using two monoclonal antibody clones

**DOI:** 10.1371/journal.pgph.0007564

**Published:** 2026-11-03

**Authors:** Sarah Gwyn, Karana Wickens, Helena Brazal Monzo, Chris Drakeley, Stephane Pelleau, Michael White, Zachary Reynolds, Henry Kanyi, Richelle W. Kihoro, Henry Emisiko, Sammy M. Njenga, Diana L. Martin

**Affiliations:** 1 Division of Parasitic Diseases and Malaria, National Center for Zoonotic, Waterborne, and Environmental Diseases, Centers for Disease Control and Prevention, Atlanta, Georgia, United States of America; 2 Department of Immunology, Faculty of Infectious and Tropical Diseases, London School of Hygiene and Tropical Medicine, London, United Kingdom; 3 Infectious Disease Epidemiology and Analytics Unit, Department of Global Health, Institut Pasteur, Université Paris-Cité, Paris, France; 4 Synergy America, Inc., Atlanta, Georgia, United States of America; 5 Eastern and Southern Africa Centre of International Parasite Control, Kenya Medical Research Institute, Nairobi, Kenya; University of Nebraska Medical Center, UNITED STATES OF AMERICA

## Abstract

The bacterium *Chlamydia trachomatis* causes three distinct conditions – urogenital chlamydia, the eye disease trachoma, and lymphogranuloma venereum. Use of serological testing for exposure to *C. trachomatis* for evaluating ocular disease risk factors and population-based surveillance has expanded over the past decade. We developed chimeric monoclonal antibodies to the *C. trachomatis* antigen Pgp3 as standardized reagents for Pgp3 serological assays. Here we use two anti-Pgp3 monoclonals (1G1 and 1C3) to conduct an interlaboratory comparison of Pgp3 Luminex-based multiplex bead serological assays in 4 different laboratories (each with their own protocol) with 5 operators using 3 different instrument types. Antibodies spotted onto filter paper were stable for two weeks at room temperature. Accuracy and precision were evaluated at 3 concentrations for each monoclonal (high, medium, and low). Accuracy within 20% was achieved for 11/21 conditions for 1G1 and 18/21 conditions for 1C3; the high concentration resulted in the poorest accuracy. Precision was within 10% for all low and medium concentrations and was above 10% for several conditions at the high concentration. The fluorescence values differed between instruments but showed no difference when extrapolated to concentrations using a standard curve. These data suggest that 1C3 may be the more reliable standard reagent and that the Pgp3 multiplex assays are highly robust when comparing different instruments and assay protocols.

## Introduction

*Chlamydia trachomatis* can lead to different clinical conditions depending on the route of infection and infecting serovars [1]. Serovars A-C cause trachoma, an eye disease that can cause blindness through scarring of the upper tarsal conjunctiva and loss of elasticity of the upper lid due to repeated conjunctival infections. Serovars D-K cause urogenital chlamydia infection via sexual transmission and can progress to pelvic inflammatory disease, infertility, and ectopic pregnancy in females. Serovars L1-L3 cause lymphogranuloma venereum (LGV) primarily associated with urogenital or anorectal infection. Infection is diagnosed with nucleic acid amplification tests; serology is increasingly used for surveillance or to identify risk factors for progressive diseases [2].

Most serological tests currently in use for *C. trachomatis* target the antigen Pgp3. Pgp3-based assays have been developed for multiplex bead-based immunoassays (MBA), enzyme-linked immunosorbent assays (ELISA) and rapid lateral flow assays (LFA). Several studies have assessed the association between Pgp3 serology and risk of tubal factor infertility and pelvic inflammatory diseases [3–6], ovarian cancer [7–11], and miscarriage [12]. Pgp3 serology has also been used to estimate population-level exposure to *C. trachomatis* for urogenital chlamydia [3,13–15].

Serological surveillance has been increasingly employed by national trachoma programs to better understand ocular *C. trachomatis* exposure in areas of persistent and recrudescent trachoma, as well as a potential tool for post-validation surveillance [16–21]. Pgp3-MBA [22] and Pgp3-LFA [23] methods used herein were developed specifically for use in trachoma programs, with the Pgp3-LFA offering a low-tech, low-cost option, and the Pgp3-MBA offering an option for use in integrated serosurveys. These have been compared in several population-based surveys [24–27]. The generation of mouse-human chimeric monoclonal antibodies (mAb) allowed a full validation of test performance [28]. Two separate clones (1G1 and 1C3) indicated that both assays have a limit of detection (LOD) of 75 ng/mL(28). Clones were stable when stored at -20°C and 4°C for up to 2 years; however, warmer temperatures that might be encountered when shipping reagents were not assessed [28]. While the LFA is a qualitative assay with a standardized protocol, the MBA is a semi-quantitative assay with methods and instrumentation varying between laboratories conducting integrated serosurveillance that independently developed assays. Here we present data evaluating the inter-laboratory robustness of the Pgp3-MBA using both Pgp3 mAb clones run in 4 laboratories utilizing 3 instrument types and assay protocols.

## Methods

### Stability testing

Pgp3 mAb clones 1G1 and 1C3 were diluted in normal human serum (NHS) to generate concentrations ranging from 62.5 to 4000 ng/mL. Five µL of each concentration was spotted onto a 6 mm circular extension of TropBio filter paper (TropBio Pty Ltd., Townsville, Queensland, Australia). Extensions are calibrated to absorb 10 µL of whole blood; therefore, we spotted 5 µL of serum assuming a 50% hematocrit. Specimens were allowed to air-dry for at least 2 hours at room temperature (RT) (20–24°C) and then stored in sealed plastic bags with desiccant at -20°C for 2 weeks prior to starting the stability time course.

At day 0 of the time course, specimens (n = 8 per condition) were placed at both RT and in a 37°C incubator. Designated specimens were transferred to a -20°C freezer at days 3, 7, and 21 until testing. Baseline samples remained at -20°C throughout the duration of the time course. Samples were prepared and tested by multiplex bead assay (MBA) at CDC on a Bio-Plex 200® instrument.

### Interlaboratory comparison

Pgp3 mAb clones 1G1 and 1C3 were diluted in NHS to create 12-point standard curves with two-fold dilutions with final concentrations of 8–16,000 ng/mL. For each mAb clone, 3 samples representing a high, medium and low concentration (6000, 1200 and 200 ng/mL) were created by spiking mAb into NHS and treated as unknowns. All samples were spotted (5 µL/extension) onto TropBio filter paper. Specimens were air-dried for at least 2 hours at RT and stored in sealed plastic bags with desiccant at -20°C until testing (at CDC) or shipping (to external laboratories).

Samples were shipped to participating laboratories at RT and were stored at -20°C until testing. Samples were tested on 3–5 different days in 4 different laboratories, by 5 different operators and by 3 different instruments (Table 1). Each laboratory used its laboratory-specific protocols, as described below. Each laboratory also generated its own antigen-coupled beads, with the exception of Lab B, which used antigen-couple beads previously provided by Lab A. Table 2 highlights the key technical differences between the protocols.

**Table 1:** Laboratories, operators and instrument types for testing.

Lab	Operator	Instrument
**A**	1	Bio-Plex 200
**A**	2	Bio-Plex 200
**A**	1	MAGPIX
**A**	2	MAGPIX
**B**	3	Bio-Plex 200
**C**	4	MAGPIX
**D**	5	Intelliflex

**Table 2:** Technical differences between laboratory assay protocols.

Step	Lab A	Lab B	Lab C	Lab D
**Secondary reagents**	Anti-human IgG + IgG4 and SAPE added in separate steps		Anti-IgG directly conjugated to PE and added in one step	
**Sample incubation**	90-minute incubation			30-minute incubation
**Secondary incubation**	45 minutes anti-IgG/IgG4-biotin 30 minutes SAPE		Anti-IgG-PE for 90 minutes	Anti-IgG-PE for 15 minutes
**Buffer incubation wash step**	30-minute incubation with Buffer A			None

SAPE = streptavidin phycoerythrin; PE = phycoerythrin

Labs A and B followed the protocol previously described [29]. Briefly, samples were diluted to a final serum concentration of 1:400 in Buffer B (1X PBS, 0.5% PVA, 0.8% PVP, 0.02% sodium azide, 0.3% Tween-20, 0.5% casein, 3 µg/mL *E. coli* extract) and stored at 4°C for at least one day. Diluted sample was incubated with Pgp3-coupled MagPlex microspheres (Luminex, Austin, TX US) for 90 minutes. Beads were washed 3x with PBST (1X PBS, 0.05% Tween-20) and incubated with biotinylated mouse anti-human IgG and biotinylated mouse anti-human IgG4 for 45 minutes. After three additional washes with PBST, beads were incubated for 30 minutes with phycoerythrin-labeled streptavidin (SAPE). Beads were washed three times with PBST and incubated for 30 minutes with Buffer A (PBST, 0.5% BSA, 0.02% sodium azide) to remove loosely bound antibodies. After one more wash with PBST, beads were resuspended in 1X PBS, and stored overnight at 4°C. The next day, beads were read on either a Bio-Plex 200 instrument (Bio-Rad, Hercules, CA, USA) (Lab A and Lab B) or a MAGPIX instrument (Luminex) (Lab A).

Lab C followed their previously published protocol [30]. Samples were diluted to 1:400 in Buffer B2 (1X PBS, 0.05% Tween-20, 0.1% casein, 0.5% PVA, 0.5% PVP, 0.02% sodium azide, 15.25 µg/mL *E. coli* extract) and stored at 4°C overnight. A 50 µL aliquot of the bead mixture was added to each well, followed by washing with PBST (PBS + 0.05% Tween-20). Diluted sample was incubated with Pgp3-coupled MagPlex microspheres (Luminex) for 90 minutes. Beads were washed 3x with PBST and incubated with anti-human IgG directly conjugated to PE for 90 minutes. Beads were washed 3x with PBST, and incubated with Buffer A (1X PBS, 0.05% Tween-20, 0.5% BSA, 0.02% sodium azide) for 30 minutes. After one more wash with PBST, beads were suspended in 1X PBS and read immediately on a MAGPIX instrument (Luminex).

The protocol for Lab D has been described elsewhere [31]. Samples were diluted to 1:400 in Buffer B2 and stored at 4°C for at least one day. Samples were incubated vol/vol with Pgp3-coupled MagPlex microspheres (Luminex) for 30 minutes, washed 3 times with PBST (1X PBS, 1% BSA, 0.05% Tween-20) and incubated with goat anti-human IgG conjugated to PE for 15 minutes. After 3 more washes with PBST, beads were suspended in 100uL PBST, put on a shaker for 5 minutes and read on the Intelliflex (Luminex), with acquisition mode Luminex 200, low PMT.

### Analysis

All data were reported as the median fluorescence intensity (MFI) with background subtracted (MFI-bg). For stability testing, the percent change (100x [{final MFI – original MFI} / original MFI]) was determined for each clone between baseline (-20°C) and each time point at each temperature.

For the inter-laboratory comparison, the calculated concentration (in ng/mL) of each unknown sample for each clone was interpolated in GraphPad Prism 9 (GraphPad Software, CA, USA) by log transforming the average values of the standard curve samples and fitting with 5-parameter logistic equations. These interpolated values were used in the analysis. Linear regression was used to compare the MFI-bg after storage with baseline MFI-bg. The coefficient of determination (R squared) and best-fit slope were calculated for each clone, temperature, and timepoint using GraphPad Prism 9 (GraphPad Software, CA, USA).

The accuracy was determined as the calculated concentration / actual concentration x 100. The desired range for accuracy was 80*–*120%. Within-laboratory precision was determined as the average percent coefficient of variation (%CV) between calculated concentrations of unknown samples run on different plates within the same laboratory. Inter-laboratory precision was determined as the mean percent coefficient of variation (%CV) between calculated concentrations of unknown samples run on different plates between different labs. Definitions are derived from the International Council for Harmonisation of Technical Requirements for Pharmaceuticals for Human Use (ICH_Q2-R2_Document_Step2_Guideline_2022_0324.pdf).

To evaluate percent recovery independent of direction, absolute error was defined as the absolute difference of percent recovery from 100%. Differences in absolute error between clones were assessed using a stratified Wilcoxon-Mann-Whitney test (Van Elteren test), with concentration as the stratification factor [32] using the coin package in R (R Core Team, 2023). Accuracy relative to the desired range of 80–120% recovery was evaluated using McNemar’s test for paired binary data, with exact p-values reported [33]. Inter-laboratory precision was quantified as the absolute difference between each unique testing condition’s mean interpolated concentration and the corresponding group mean. Differences in inter-laboratory precision between monoclonals were compared using a Van Elteren test, with concentration as the stratification factor.

Because ‘acceptance’ is a binary outcome and is assessed from paired measurements within each laboratory, differences were evaluated using McNemar’s test for paired data. Percent recovery and inter-laboratory precision were used to summarize overall assay performance across labs rather than quantify within-lab differences. Labs were treated as independent units and comparisons were performed using stratified rank-based tests.

## Results

### Stability

The coefficient of determination and slope for each clone comparing baseline -20°C storage and each timepoint and temperature are shown in Fig 1. For clone 1G1, the R squared value remained greater than 0.997 for all timepoints and temperatures; the slope decreased over time, with the lowest slope being 0.508 at 37°C at 21 days. For clone 1C3, the R squared value ranged from 0.974 to 0.999 and the slope remained above 0.902 at all time points except for 21 days at 37°C, where the slope was 0.718.

**Fig 1:**
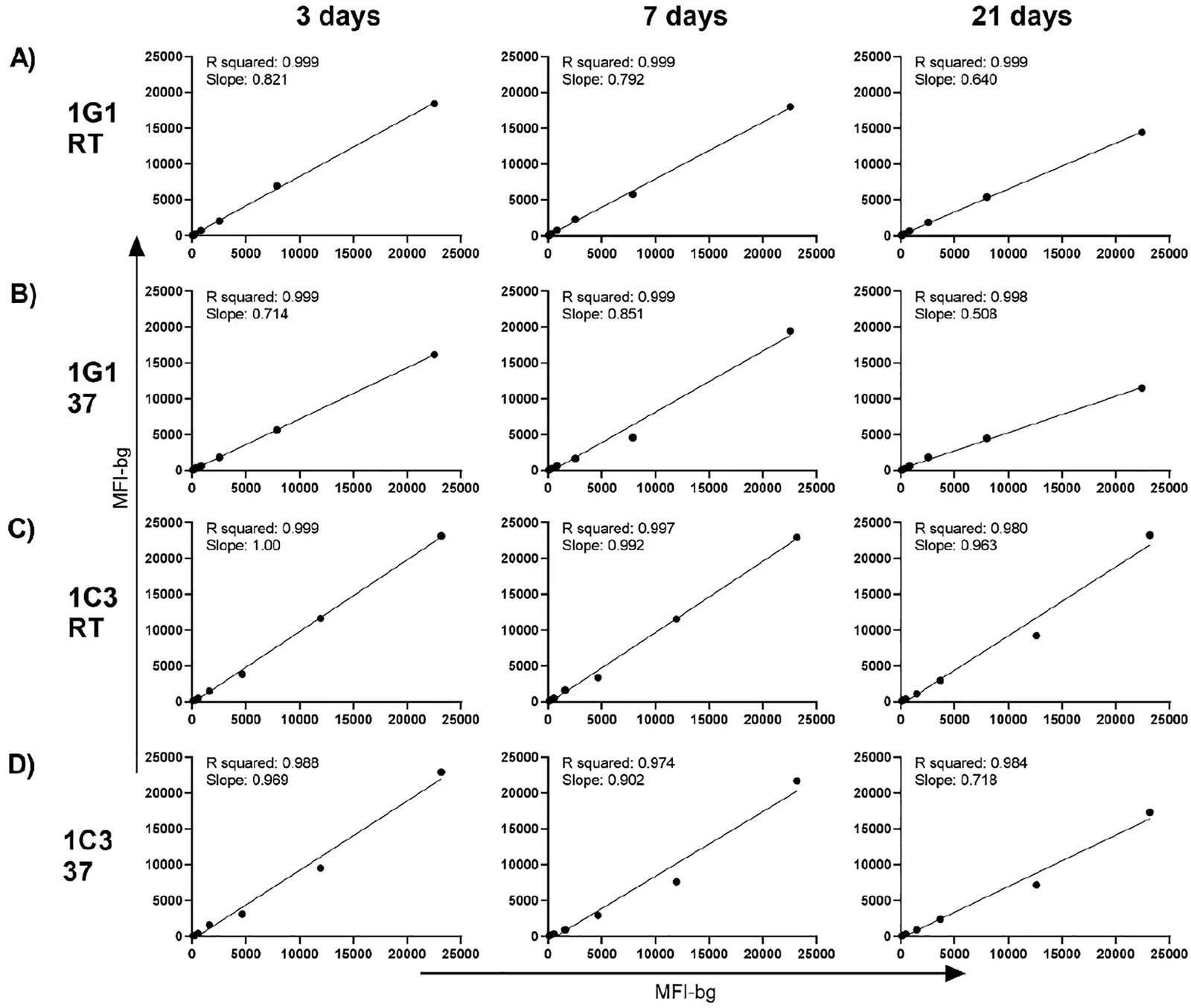
Stability of anti-Pgp3 antibody clones 1G1 and 1C3 at RT and 37°C for 3, 7 and 21 days. The coefficients of determination between MFI-bg (median fluorescence intensity minus buffer alone) of baseline samples (stored at -20°C) (X axis) and MFI-bg of samples stored at designated temperature (Y axis) are shown for each time point for **A)** Clone 1G1 at RT (room temperature) **B)** Clone 1G1 at 37°C **C)** Clone 1C3 at RT and **D)** Clone 1C3 at 37°C.

### Interlaboratory comparison

The calculated concentrations for each unknown in each laboratory are shown in Fig 2. The accuracy was in the desired range of 80–120% for the medium and low positive unknowns for clone 1C3 in all labs (Table 3). Clone 1C3 had fewer out of range results than clone 1G1 (3/21 vs 10/21) (p = 0.039). 1C3 also showed lower absolute error than 1G1 (p = 0.038). For both measures, differences were driven primarily by results at the lowest concentration, with limited differences observed at higher concentrations (Table 3). Raw MFI-bg values for standard curves are shown in S1 Fig.

**Table 3:** Accuracy of Pgp3 multiplex assays in four laboratories using high, medium, and low monoclonal antibody concentrations.

Lab		A				B	C	D
Instrument		BP		MP		BP	MP	IF
Clone	Conc (ng/mL)	Operator 1	Operator 2	Operator 1	Operator 2	Operator 3	Operator 4	Operator 5
1G1	200	75%	87%	78%	79%	77%	103%	78%
	1200	80%	90%	86%	94%	92%	121%	107%
	6000	79%	81%	89%	97%	66%	129%	38%
1C3	200	107%	112%	101%	111%	112%	114%	106%
	1200	88%	92%	96%	93%	93%	99%	87%
	6000	67%	82%	77%	114%	70%	112%	104%

Accuracy = calculated concentration/expected concentration x 100. BP = Bio-Plex; MP = MAGPIX, IF = Intelliflex.

**Fig 2:**
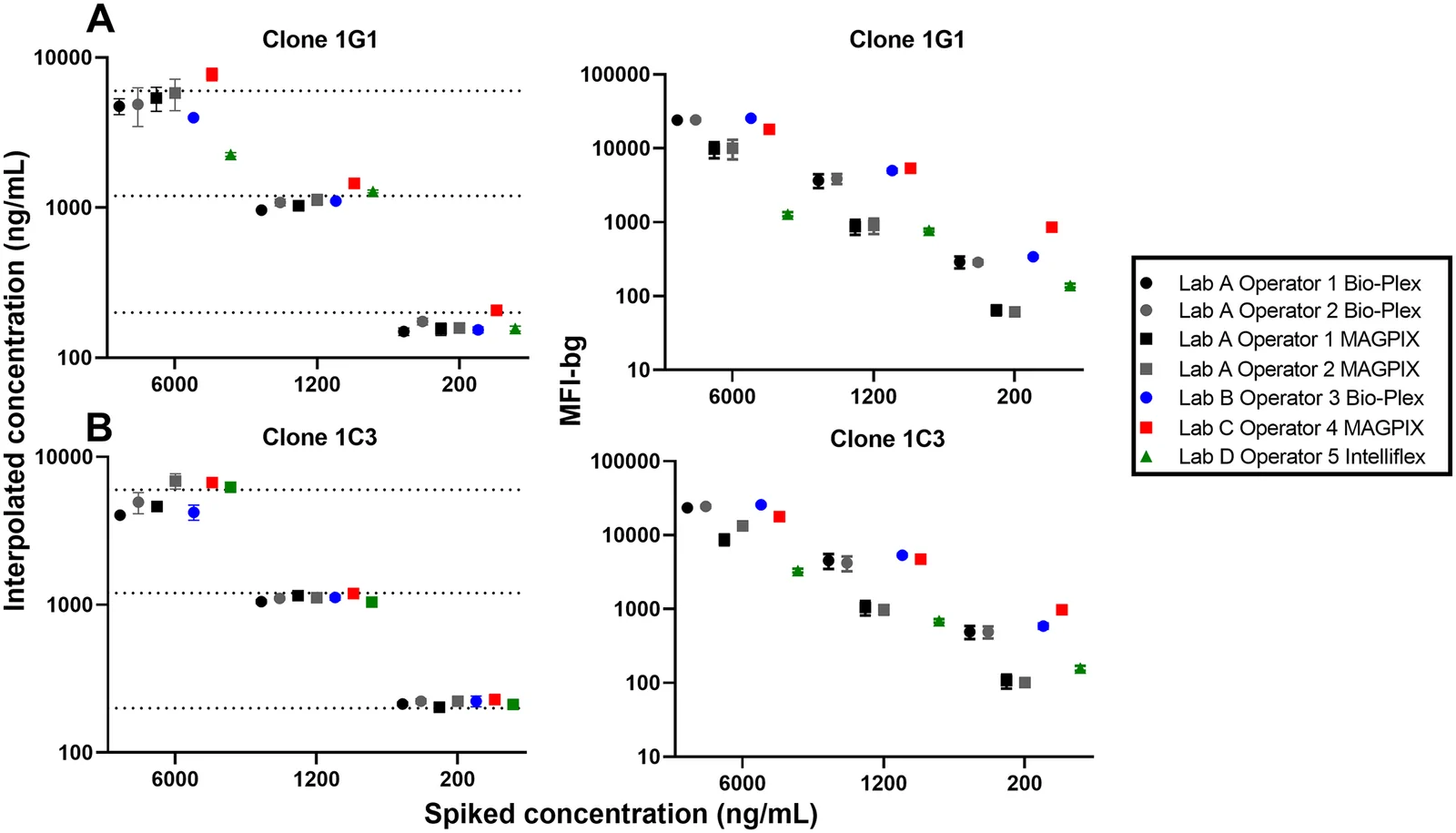
Calculated concentration and MFI-bg of each unknown sample compared to known concentration for 1G1 and 1C3. Each point represents the average calculated concentration across multiple days of testing. Error bars represent standard deviation. The dotted line is at the actual concentration.

Within-laboratory and inter-laboratory precision for the three unknown samples for each clone are shown in Table 4. Percent CVs were below 10 for each clone for all labs for medium and low positive samples for within-laboratory precision. For inter-laboratory precision, percent CVs were below 15 for low and medium positive samples but were above 20 for high positive samples for each clone. Inter-laboratory precision differed between monoclonals when laboratory-level mean concentrations were compared, with clone 1C3 showing lower deviation from the group mean than clone 1G1 (p = 0.022).

**Table 4:** The percent coefficient of variation for within laboratory precision (%CV_WL_) and inter-laboratory precision (%CV_IL_) is shown for each clone in each laboratory with a high, medium and low positive sample. BP = Bio-Plex; MP = MAGPIX, IF = Intelliflex.

	Lab	A				B	C	D	All
	Instrument	BP		MP		BP	MP	IF	All
	Operator	1	2	1	2	3	4	5	All
**Clone**	**Conc (ng/mL)**	%CV_WL_	%CV_WL_	%CV_WL_	%CV_WL_	%CV_WL_	%CV_WL_	%CV_WL_	%CV_IL_
1G1	200	5.3	4.1	8.5	2.5	4.0	2.2	3.2	11.3
	1200	6.2	4.2	6.3	6.7	1.6	4.5	1.8	13.3
	6000	10.9	23.7	16.3	19.3	1.5	7.8	2.4	31.3
1C3	200	6.7	5.6	6.6	5.7	7.3	3.2	2.5	3.8
	1200	6.5	3.8	6.5	8.9	3.5	6.1	1.0	4.4
	6000	7.0	23.1	8.3	16.8	10.1	5.1	1.4	20.6

## Discussion

Our findings demonstrate the robustness of the Pgp3-MBA across a range of conditions and assay permutations used by different laboratories. While the different instrument types and protocols yielded different raw data MFI readouts, the data extrapolated using the Pgp3 mAb standard curve showed high precision between laboratories. These data suggest that Pgp3 mAb can be used to standardize Pgp3 antibody results between labs utilizing different multiplex bead assay (MBA) protocols and instrumentation. Alternatively, unadjusted data classified as positive or negative based on cutoffs determined for each instrument and protocol should yield reproducible data.

This study also evaluated the real-world performance of the two different clones following shipment to laboratories on multiple continents. There were differences in stability and performance between the two clones evaluated. Clone 1C3 was stable for 21 days at room temperature, while signal began to decrease for clone 1G1 after three days at room temperature. These results suggest that clone 1C3 does not require cold storage for transportation lasting under 21 days and may be more suitable than 1G1 for transferring to different laboratories. There were also quantitative differences in performance both between the two clones and between samples with different levels of antibody. Clone 1C3 had accuracy within the desired range of 80–120% for more testing situations (18/21) than clone 1G1 (11/21). For 1C3, the 3 readings outside of the desired range were with the high positive unknown samples. This should not be an issue for trachoma programs, or population-based surveys in general, as results are typically interpreted as positive or negative so obtaining accurate values closer to the cutoff are more critical. Additionally, the ranges chosen as acceptable were derived from the International Council for Harmonisation’s acceptance criterion for accuracy in pharmaceutical analyses, which may be more stringent than for a serological test. The percent CVs for both clones were all within range, except for the inter-laboratory CV for the high positive sample for clone 1G1. These data indicate that clone 1C3 yields more accurate and reproducible results than clone 1G1.

There are some limitations to this work. This evaluation was only done with one lot of Pgp3 mAb. Additional lots will need to be tested to ensure there is consistent performance between lots across different laboratories. Next, because Luminex-based instruments used here are optimized for multi-pathogen testing, additional next steps will be required to evaluate this performance within the context of additional antigens as part of a monoplex versus multiplex approach. Finally, we used the standard curve to normalize between laboratories, but these tests are not operationally quantitative, therefore the data shown here are not necessarily reflective of what would normally be the machine output. Next steps could investigate using the mAb to generate limit of quantitation using different instruments and assays and comparing prevalence data from a common specimen set.

mAb such as the ones described here have a number of applications. First, validation for serological tests can be difficult due to the lack of standardized reagents and controls. Sera from actively infected persons/patients can be useful, but sera generally remain antibody-positive long after infection clears, and longitudinal data show that persons with infection tend to have higher antibody levels than persons whose infection has cleared [29]. Using the mAb allows us to evaluate the test performance across the range of the assay and determine the accuracy and linearity of a test. Next, this reagent could be useful for evaluating new tests, transferring technology to new labs and training and competency testing. This reagent could also be used to develop proficiency testing panels to ensure assay performance across different laboratories by virtue of having standardized concentrations. The approach described here can be generalized to the growing field of multi-pathogen serosurveillance, for which qualified reagents such as the Pgp3 mAb will be critical for assay standardization across multiple sites.

### Disclaimer

The findings and conclusions in this report are those of the authors and do not necessarily represent the official position of the Centers for Disease Control and Prevention.

## Supporting information

S1 Fig MFI-bg by lab for standard curve samples for 1G1 (left) and 1C3 (right). Error bars represent standard deviation. MFI-bg = median fluorescence intensity minus background (buffer alone).
(TIF)

S1 Table Dataset. (XLSX)

## References

[1] Hocking JS, Geisler WM, Kong FYS. Update on the epidemiology, screening, and management of *Chlamydia trachomatis* infection. Infect Dis Clin North Am. 2023;37(2):267–88. 10.1016/j.idc.2023.02.007 37005162

[2] Woodhall SC, Gorwitz RJ, Migchelsen SJ, Gottlieb SL, Horner PJ, Geisler WM, et al. Advancing the public health applications of *Chlamydia trachomatis* serology. Lancet Infect Dis. 2018;18(12):e399–407. doi: 10.1016/S1473-3099(18)30159-2 29983342 PMC6414067

[3] Anyalechi GE, Hong J, Danavall DC, Martin DL, Gwyn SE, Horner PJ. High Plasmid Gene Protein 3 (Pgp3) *Chlamydia trachomatis* seropositivity, pelvic inflammatory disease, and infertility among women, national health and nutrition examination survey, United States, 2013-2016. Clin Infect Dis. 2021;73(8):1507–16. 10.1093/cid/ciab506 PMC8674123 34050737

[4] Anyalechi GE, Hong J, Kirkcaldy RD, Wiesenfeld HC, Horner P, Wills GS, et al. Chlamydial Pgp3 seropositivity and population-attributable fraction among women with tubal factor infertility. Sex Transm Dis. 2022;49(8):527–33. doi: 10.1097/OLQ.0000000000001434 34110735 PMC9208281

[5] Holster T, Joki-Korpela P, Yu H, Brunham RC, Tiitinen A, Paavonen J, et al. Serum IgG1 and IgG3 antibody responses to *Chlamydia trachomatis* Pgp3 and Hsp60 in tubal factor infertility. J Infect Dis. 2025;231(6):e1057–64. doi: 10.1093/infdis/jiaf092 39981649 PMC12247805

[6] Li P, Chen Z. Association between serum *Chlamydia trachomatis* antibody levels and infertility among reproductive-aged women in the U.S. Front Public Health. 2023;11:1117245. doi: 10.3389/fpubh.2023.1117245 37089503 PMC10113615

[7] NCDC and NIMR release findings of COVID-19 household seroprevalence surveys in four states of Nigeria.

[8] Fortner RT, Terry KL, Bender N, Brenner N, Hufnagel K, Butt J, et al. Sexually transmitted infections and risk of epithelial ovarian cancer: Results from the Nurses’ Health Studies. Br J Cancer. 2019;120(8):855–60. doi: 10.1038/s41416-019-0422-9 30894687 PMC6474309

[9] Idahl A, Le Cornet C, González Maldonado S, Waterboer T, Bender N, Tjønneland A, et al. Serologic markers of *Chlamydia trachomatis* and other sexually transmitted infections and subsequent ovarian cancer risk: Results from the EPIC cohort. Int J Cancer. 2020;147(8):2042–52. doi: 10.1002/ijc.32999 32243586

[10] Ness RB, Goodman MT, Shen C, Brunham RC. Serologic evidence of past infection with *Chlamydia trachomatis*, in relation to ovarian cancer. J Infect Dis. 2003;187(7):1147–52. doi: 10.1086/368380 12660930

[11] Trabert B, Waterboer T, Idahl A, Brenner N, Brinton LA, Butt J, et al. Antibodies against *Chlamydia trachomatis* and ovarian cancer risk in two independent populations. J Natl Cancer Inst. 2019;111(2):129–36. doi: 10.1093/jnci/djy084 29790947 PMC6376903

[12] Horne AW, Wheelhouse N, Horner PJ, Duncan WC. Association of past *Chlamydia trachomatis* infection with miscarriage. JAMA Netw Open. 2020;3(10):e2018799. doi: 10.1001/jamanetworkopen.2020.18799 33026447 PMC7542304

[13] Mazraani R, Timms P, Hill PC, Suaalii-Sauni T, Niupulusu T, Temese SVA, et al. Evaluation of a PGP3 ELISA for surveillance of the burden of Chlamydia infection in women from Australia and Samoa. Pathog Dis. 2019;77(3):ftz031. doi: 10.1093/femspd/ftz031 31201421 PMC6607412

[14] Shui J, Xie D, Zhao J, Ao C, Lin H, Liang Y, et al. Seroepidemiology of *Chlamydia trachomatis* infection in the general population of Northern China: The Jidong community cohort study. Front Microbiol. 2021;12:729016. doi: 10.3389/fmicb.2021.729016 34650533 PMC8507574

[15] Woodhall SC, Wills GS, Horner PJ, Craig R, Mindell JS, Murphy G, et al. *Chlamydia trachomatis* Pgp3 antibody population seroprevalence before and during an Era of widespread opportunistic Chlamydia Screening in England (1994-2012). PLoS One. 2017;12(1):e0152810. doi: 10.1371/journal.pone.0152810 28129328 PMC5271337

[16] Nesemann JM, Morocho-Alburqueque N, Quincho-Lopez A, Wittberg DM, Honorio-Morales HA, Durand S. Discrepancy between active trachoma and tests of chlamydial infection in Alto Amazonas, Peru. Clin Infect Dis. 2025. 10.1093/cid/ciaf506 PMC12959316 40971876

[17] Lynn MK, Ayele Z, Chernet A, Goodhew EB, Wickens K, Sata E, et al. Prevalence of ocular *Chlamydia trachomatis* infection and antibodies within districts persistently endemic for trachoma, Amhara, Ethiopia. PLoS Negl Trop Dis. 2025;19(3):e0012900. doi: 10.1371/journal.pntd.0012900 40067808 PMC11936273

[18] Sitoe HM, Oswald WE, Zita F, Fall M, Momade T, Adams MW, et al. Ongoing transmission of trachoma in low prevalence districts in Mozambique: Results from four cross-sectional enhanced impact surveys, 2022. Sci Rep. 2024;14(1):22842. doi: 10.1038/s41598-024-71201-z 39406720 PMC11480103

[19] Sanders AM, Elshafie BE, Abdalla Z, Simmons C, Goodhew EB, Gonzalez TA, et al. Serological responses to trachoma antigens prior to the start of mass drug administration: Results from population-based baseline surveys, North Darfur, Sudan. Am J Trop Med Hyg. 2024;111(3_Suppl):49–57. doi: 10.4269/ajtmh.23-0608 38507810 PMC11374501

[20] Atekem K, Harding-Esch EM, Martin DL, Downs P, Palmer SL, Kaboré A, et al. High prevalence of trachomatous inflammation-follicular with no trachomatous trichiasis: Can alternative indicators explain the epidemiology of trachoma in Côte d’Ivoire?. Int Health. 2023;15(15 Suppl 2):ii3–11. doi: 10.1093/inthealth/ihad069 38048384 PMC10695457

[21] Hammou J, Guagliardo SAJ, Obtel M, Razine R, Haroun AE, Youbi M, et al. Post-validation survey in two districts of Morocco after the elimination of trachoma as a public health problem. Am J Trop Med Hyg. 2022;106(5):1370–8. doi: 10.4269/ajtmh.21-1140 35344929 PMC9128706

[22] Goodhew EB, Priest JW, Moss DM, Zhong G, Munoz B, Mkocha H, et al. CT694 and pgp3 as serological tools for monitoring trachoma programs. PLoS Negl Trop Dis. 2012;6(11):e1873. doi: 10.1371/journal.pntd.0001873 23133684 PMC3486877

[23] Gwyn S, Mitchell A, Dean D, Mkocha H, Handali S, Martin DL. Lateral flow-based antibody testing for *Chlamydia trachomatis*. J Immunol Methods. 2016;435:27–31. doi: 10.1016/j.jim.2016.05.008 27208400

[24] Gwyn S, Awoussi MS, Bakhtiari A, Bronzan RN, Crowley K, Harding-Esch EM, et al. Comparison of platforms for testing antibodies to *Chlamydia trachomatis* antigens in the Democratic Republic of the Congo and Togo. Sci Rep. 2021;11(1):7225. doi: 10.1038/s41598-021-86639-8 33790370 PMC8012353

[25] Gwyn S, Cooley G, Goodhew B, Kohlhoff S, Banniettis N, Wiegand R, et al. Comparison of platforms for testing antibody responses against the *Chlamydia trachomatis* antigen Pgp3. Am J Trop Med Hyg. 2017;97(6):1662–8. doi: 10.4269/ajtmh.17-0292 29016320 PMC5805053

[26] Nash SD, Astale T, Nute AW, Bethea D, Chernet A, Sata E, et al. Population-based prevalence of *Chlamydia trachomatis* infection and antibodies in four districts with varying levels of Trachoma endemicity in Amhara, Ethiopia. Am J Trop Med Hyg. 2021;104(1):207–15. doi: 10.4269/ajtmh.20-0777 33200728 PMC7790060

[27] Kamau E, Ante-Testard PA, Gwyn S, Blumberg S, Abdalla Z, Aiemjoy K, et al. Characterizing trachoma elimination using serology. Nat Commun. 2025;16(1):5545. doi: 10.1038/s41467-025-60581-z 40593530 PMC12216694

[28] Goodhew B, Tang X, Goldstein J, Lee J, Martin D, Gwyn S. Validation of immunoassays for the *Chlamydia trachomatis* antigen Pgp3 using a chimeric monoclonal antibody. Sci Rep. 2023;13(1):7281. doi: 10.1038/s41598-023-33834-4 37142607 PMC10160048

[29] Goodhew EB, Taoaba R, Harding-Esch EM, Gwyn SE, Bakhtiari A, Butcher R, et al. Changes in trachoma indicators in Kiribati with two rounds of azithromycin mass drug administration, measured in serial population-based surveys. PLoS Negl Trop Dis. 2023;17(7):e0011441. doi: 10.1371/journal.pntd.0011441 37418501 PMC10355439

[30] Wu L, Hall T, Ssewanyana I, Oulton T, Patterson C, Vasileva H, et al. Optimisation and standardisation of a multiplex immunoassay of diverse *Plasmodium falciparum* antigens to assess changes in malaria transmission using sero-epidemiology. Wellcome Open Res. 2020;4:26. doi: 10.12688/wellcomeopenres.14950.2 32518839 PMC7255915

[31] Rosado J, Pelleau S, Cockram C, Merkling SH, Nekkab N, Demeret C, et al. Multiplex assays for the identification of serological signatures of SARS-CoV-2 infection: An antibody-based diagnostic and machine learning study. Lancet Microbe. 2021;2(2):e60–9. doi: 10.1016/S2666-5247(20)30197-X 33521709 PMC7837364

[32] Hothorn LA. Multiple comparisons and multiple contrasts in randomized dose-response trials--confidence interval oriented approaches. J Biopharm Stat. 2006;16(5):711–31. doi: 10.1080/10543400600860576 17037267

[33] Agresti A, Min Y. Effects and non-effects of paired identical observations in comparing proportions with binary matched-pairs data. Stat Med. 2004;23(1):65–75. doi: 10.1002/sim.1589 14695640

