# Supplementary material for: Interlaboratory comparison of a multiplex bead assay against the Chlamydia trachomatis antigen Pgp3 using two monoclonal antibody clones
Source: PLOS Glob Public Health. 2026 Nov 3;6(11):e0007564. doi: 10.1371/journal.pgph.0007564 (PMC13730333; doi:10.1371/journal.pgph.0007564)
Supplement: Supplementary file 2 [file pgph.0007564.s005.docx]

PGPH-D-26-01680R1
Interlaboratory comparison of a multiplex bead assay against the Chlamydia trachomatis antigen Pgp3 using two monoclonal antibody clones
PLOS Global Public Health

Dear Dr. Martin,

Thank you for submitting your manuscript to PLOS Global Public Health. After careful consideration, we feel that it has merit but does not fully meet PLOS Global Public Health’s publication criteria as it currently stands. Therefore, we invite you to submit a revised version of the manuscript that addresses the points raised during the review process.

Please find some minor suggestions from the reviewers below. Please revise your manuscript to address these comments, providing a point-by-point response to the reviewers upon resubmission.

………………………………..

6. Review Comments to the Author

Please use the space provided to explain your answers to the questions above. You may also include additional comments for the author, including concerns about dual publication, research ethics, or publication ethics. (Please upload your review as an attachment if it exceeds 20,000 characters)

**Reviewer #1:** Line 122:
How was the correlation coefficient calculated? Please note this information here.

**Response: This has been edited to correct that we calculated coefficient of determination, not correlation coefficient, with the additional information provided.**

Line 166:
The correlation coefficient has already been noted for each standard curve in Figure 1 for each clone as 0.999, etc. This statement in the Figure 1 legend is now unclear. Do the authors mean the residuals? Please revise.

**Response: We have edited the manuscript to reflect that the coefficient of determination rather than the correlation coefficient was calculated.**

**Reviewer #2:** Please review for minor grammatical errors (e.g. lines 126 and 174).

**Response: We have reviewed the manuscript again for grammatical or other errors.**
