# Supplementary material for: Interlaboratory comparison of a multiplex bead assay against the Chlamydia trachomatis antigen Pgp3 using two monoclonal antibody clones
Source: PLOS Glob Public Health. 2026 Nov 3;6(11):e0007564. doi: 10.1371/journal.pgph.0007564 (PMC13730333; doi:10.1371/journal.pgph.0007564)
Supplement: Supplementary file 1 [file pgph.0007564.s004.docx]

PGPH-D-26-01680
Interlaboratory comparison of a multiplex bead assay against the Chlamydia trachomatis antigen Pgp3 using two monoclonal antibody clones
PLOS Global Public Health

Dear Dr. Diana L. Martin,

Thank you for submitting your manuscript to PLOS Global Public Health. After careful consideration, we feel that it has merit but does not fully meet PLOS Global Public Health’s publication criteria as it currently stands - minor revisions required. Therefore, we invite you to submit a revised version of the manuscript that addresses the points raised during the review process.

==============================

The authors have done an excellent job at evaluating a novel multiplex bead assay using two monoclonal antibody clones targeting the C. trachomatis antigen, Pgp3. Very minor revisions required as indicated by the reviewers below, in addition to:

1) Rename Figure 2 (Line 172) as Supplementary Figure 1 and create panels A & B to indicate which antibody clone is evaluated (A: 1G1 B: 1C3).

**Response: We have added A and B to the Figure 2 but have left it as Figure 2; we believe this is critical enough to the conclusion of the paper to have it be part of the main text and not put in as a supplement. This is showing the accuracy of the assays, which is one of the key validation parameters being assessed in the manuscript.**

Supplemental Figure 1 and Figure 2 are two distinct Figures. We have created panels A & B to indicate antibody clone in Supplemental Figure 1.

2) Please ensure Table titles are listed above the Table and any footnotes, text etc. below the table

**Response: we have moved the Table titles above each table**

==============================

Please submit your revised manuscript by July 22, 2026. If you will need more time than this to complete your revisions, please reply to this message or contact the journal office at globalpubhealth@plos.org. When you're ready to submit your revision, log on to <https://www.editorialmanager.com/pgph/> and select the 'Submissions Needing Revision' folder to locate your manuscript file.

Please include the following items when submitting your revised manuscript:

- A letter that responds to each point raised by the editor and reviewer(s). You should upload this letter as a separate file labeled 'Response to Reviewers'.
- A marked-up copy of your manuscript that highlights changes made to the original version. You should upload this as a separate file labeled 'Revised Manuscript with Track Changes'.
- An unmarked version of your revised paper without tracked changes. You should upload this as a separate file labeled 'Manuscript'.

Guidelines for resubmitting your figure files are available below the reviewer comments at the end of this letter.

As the corresponding author, your ORCID iD is verified in the submission system and will appear in the published article. PLOS supports the use of ORCID, and we encourage all coauthors to register for an ORCID iD and use it as well. Please encourage your coauthors to verify their ORCID iD within the submission system before final acceptance, as unverified ORCID iDs will not appear in the published article. *Only* the individual author can complete the verification step; PLOS staff *cannot* verify ORCID iDs on behalf of authors.

We look forward to receiving your revised manuscript.

Kind regards,

Dr. Ruwandi Kariyawasam
Academic Editor
PLOS Global Public Health

**Journal Requirements:**

1. Please amend your detailed Financial Disclosure statement. This is published with the article. It must therefore be completed in full sentences and contain the exact wording you wish to be published.

i. Please clarify all sources of financial support for your study. List the grants, grant numbers, and organizations that funded your study, including funding received from your institution. Please note that suppliers of material support, including research materials, should be recognized in the Acknowledgements section rather than in the Financial Disclosure. 
ii. State the initials, alongside each funding source, of each author to receive each grant. For example: "This work was supported by the National Institutes of Health (####### to AM; ###### to CJ) and the National Science Foundation (###### to AM)."
iii. State what role the funders took in the study. If the funders had no role in your study, please state: “The funders had no role in study design, data collection and analysis, decision to publish, or preparation of the manuscript.”
iv. If any authors received a salary from any of your funders, please state which authors and which funders.

**Response: This has been added.**

2. In the online submission form, you indicated that “The minimal data set [and accompanying code, where generated] is available from the corresponding author.”.

All PLOS journals now require all data underlying the findings described in their manuscript to be freely available to other researchers, either 
1. In a public repository, 
2. Within the manuscript itself, or 
3. Uploaded as supplementary information.

This policy applies to all data except where public deposition would breach compliance with the protocol approved by your research ethics board. If your data cannot be made publicly available for ethical or legal reasons (e.g., public availability would compromise patient privacy), please explain your reasons by return email and your exemption request will be escalated to the editor for approval. Your exemption request will be handled independently and will not hold up the peer review process, but will need to be resolved should your manuscript be accepted for publication. One of the Editorial team will then be in touch if there are any issues.

**Response: We have uploaded the full dataset as Supplementary File 2 and added text to the manuscript to indicate so.**

3. We do not publish any copyright or trademark symbols that usually accompany proprietary names, eg (R), (C), or TM  (e.g. next to drug or reagent names). Please remove all instances of trademark/copyright symbols throughout the text, including Bio-Plex 200®, MAGPIX®, Intelliflex®, MagPlex®. 

**Response: We have made this correction**

If the reviewer comments include a recommendation to cite specific previously published works, please review and evaluate these publications to determine whether they are relevant and should be cited. There is no requirement to cite these works unless the editor has indicated otherwise. 

Please review your reference list to ensure that it is complete and correct. If you have cited papers that have been retracted, please include the rationale for doing so in the manuscript text, or remove these references and replace them with relevant current references. Any changes to the reference list should be mentioned in the rebuttal letter that accompanies your revised manuscript. If you need to cite a retracted article, indicate the article’s retracted status in the References list and also include a citation and full reference for the retraction notice.

5. Review Comments to the Author

Please use the space provided to explain your answers to the questions above. You may also include additional comments for the author, including concerns about dual publication, research ethics, or publication ethics. (Please upload your review as an attachment if it exceeds 20,000 characters)

**Reviewer #1:** Minor revisions:
Line 122:
The percent change is noted as (final MFI – original MFI / original MFI). The way that this is currently written indicates that the original MFI / original MFI would result in 1 and then this would be subtracted from the final MFI. Likely it should be re-written as ([final MFI – original MFI] / original MFI) x 100.

**Response: Thank you for pointing this out. The review to address your comment alerted us to the fact that we report the stability data as correlation, not percent change, so have altered the methods accordingly.**

Line 131 (and through-out):
Inter-lab precision may be better classified as reproducibility as per the ICH-Q2.

**Response: The reviewer is correct, but we did specifically didn’t use that term for a reason. Unfortunately many people in the neglected tropical disease field misuse the term “reproducibility” as simply “repeatability” (often with the same operator/assessor), and so we wanted to emphasize that we were using different laboratories. Additionally, reproducibility testing applies to validation of a specific assay – because two of the labs used their own in-house assay, we felt “reproducibility” was not the right term. However, two of the labs (Lab A and C) used the same protocol on the same instrument (BioPlex), and we have added text to indicate that this is a true reproducibility assessment as per ICH.**

Figure 1:
On the y-axis, please add the temperature of sample storage to each of the different MFI-bg’s calculated for clarity.

**Response: We have added labels on the figure for temperature, clone and days to clarify.**

**Reviewer #2:** This study evaluates the interlaboratory performance of Pgp3 luminex-based multiplex bead serological assays using two chimeric anti-Pgp3 monoclonal antibodies (1G1 and 1C3) as standardized reference reagents, across different laboratories using independently developed protocols, with different operators and instrument types. The authors suggest that Pgp3 multiplex bead assays are highly robust for comparing different instruments and assay protocols, and the 1C3 clone may be the more reliable standard reagent that yields more accurate and reproducible results.

The study addresses a clinically relevant topic, an important need in Chlamydia trachomatis serology testing and connects its use in practical applications. The study is generally well designed. A notable strength of the study is the evaluation across multiple laboratories, operators, and instrument platforms using each laboratory's routine protocol. This approach provides an assessment of real-world performance and generalizability beyond a single-site setting which strengthens the evidence supporting the reagent's robustness.

Minor comments
The methodology for the stability assessment requires further clarification. The authors state designated specimens were transferred to a -20°C freezer at days 3, 7, and 21. It is not entirely clear if all concentrations of the Pgp3 mAb clones were included in the 'designated specimens' and how it was determined which concentrations were selected for storage at the different time points. Additional methodological detail would improve reproducibility and facilitate interpretation of the stability findings.

**Response: Thank you for pointing this out. We have removed the word “designated” since all concentrations for each specimen were included in the stability testing.**

Figure 1 legend describes the stability of the clones at different storage conditions and timepoints; consider more explicit identification of clone type and post-storage timepoints in the figure panel labels to improve readability and reduce the need for cross-referencing the legend.

**Response: We have added labels on the figure for temperature, clone and days to clarify.**

The abstract notes the 1C3 clone had 11/21 conditions within the desired accuracy range and the 1G1 had 18/21 conditions, however in the results, these conditions were reported as "out of range" (10/21 vs 3/21). Later on in the discussion section, these conditions were reported as "within range". The numbers are internally consistent but flipping the framing may be confusing for the reader. Consider maintaining consistent accuracy framing for clarity.

**Response: Thank you for pointing out this discrepancy. We have updated changed the numbers in the results section to report the number of in range conditions to be consistent with the rest of the paper.**

The paper describes in detail the different laboratory-specific protocols, with Table 2 highlighting the key technical differences. Could these differences explain the inter-lab variation noted in the raw data MFI readouts? Consider discussing whether specific protocol differences contributed to the variability. This would provide additional context for interpreting the results and further strengthen the robustness assessment.

**Response: We note in lines 197-199 that both the protocols and instrument type likely contribute to the differences in raw MFI seen in the different sites. While the reviewer brings up a good point, it is one we are reluctant to address in this paper since the study was not designed to assess these differences. The BioPlex and MAGPIX were run using the exact same protocol in Lab A. The difference between these raw readouts is very similar to the differences in the raw readouts between Lab A-MAGPIX and Lab C- MAGPIX. With that very small sample size, it seems that the instrument type and protocol both affect the raw MFI. However, se would have to run all three protocols on all 3 instrument types in multiple laboratories to determine which contributed more significantly to the differences in raw MFI. Ultimately we think that is a less critical question that demonstrating that the monoclonal allows normalization between sites.**

Accuracy and precision readings outside of the desired range for the 1C3 clone were reported for the high concentration samples, but not explained. Could this be a limitation of the IC3 reagent? If not, providing a hypothesis of why this observed inaccuracy/imprecision occurred would reassure the readers it is not a limitation of the monoclonal antibody reagent.

**Response: We have added a potential explanation to line 212 for the low accuracy of high positive unknown samples.**

Overall, the manuscript is well written and easy to follow, the methodology is generally rigorous, the data is presented clearly, and the conclusions are largely supported by the data. The comments made primarily relate to clarification and expansion of the discussion rather than concerns regarding study design or data interpretation.

6. PLOS authors have the option to publish the peer review history of their article ([what does this mean?](https://track.editorialmanager.com/CL0/https:%2F%2Fjournals.plos.org%2Fglobalpublichealth%2Fs%2Feditorial-and-peer-review-process%23loc-peer-review-history/1/010f019ef325f637-f74d8dae-a391-4342-b8f2-240375490284-000000/8yZL7e2pLzywfMKRP31rr15sTL0Rxsc_ymZNDpQ5omg=258)). If published, this will include your full peer review and any attached files.


**Do you want your identity to be public for this peer review?** If you choose “no”, your identity will remain anonymous but your review may still be made public.


For information about this choice, including consent withdrawal, please see our [Privacy Policy](https://track.editorialmanager.com/CL0/https:%2F%2Fwww.plos.org%2Fprivacy-policy/1/010f019ef325f637-f74d8dae-a391-4342-b8f2-240375490284-000000/LZoFkOgmZZPqf7dl7c_L5hcS1_ZiHuXv2KYvpbQayGI=258).

Reviewer #1: **Yes:** Ellen M. E. Sykes

Reviewer #2: **Yes:** Ima Anugom

[NOTE: If reviewer comments were submitted as an attachment file, they will be attached to this email and accessible via the submission site. Please log into your account, locate the manuscript record, and check for the action link "View Attachments". If this link does not appear, there are no attachment files.]

**Figure Resubmissions:**

While revising your submission, we strongly recommend that you use PLOS’s NAAS tool (https://ngplosjournals.pagemajik.ai/artanalysis) to test your figure files. NAAS can convert your figure files to the TIFF file type and meet basic requirements (such as print size, resolution), or provide you with a report on issues that do not meet our requirements and that NAAS cannot fix.

After uploading your figures to PLOS’s NAAS tool - https://ngplosjournals.pagemajik.ai/artanalysis, NAAS will process the files provided and display the results in the "Uploaded Files" section of the page as the processing is complete. If the uploaded figures meet our requirements (or NAAS is able to fix the files to meet our requirements), the figure will be marked as "fixed" above. If NAAS is unable to fix the files, a red "failed" label will appear above. When NAAS has confirmed that the figure files meet our requirements, please download the file via the download option, and include these NAAS processed figure files when submitting your revised manuscript.
