# Supplementary material for: Interlaboratory comparison of a multiplex bead assay against the Chlamydia trachomatis antigen Pgp3 using two monoclonal antibody clones
Source: PLOS Glob Public Health. 2026 Nov 3;6(11):e0007564. doi: 10.1371/journal.pgph.0007564 (PMC13730333; doi:10.1371/journal.pgph.0007564)
Supplement: Peer review history

# Peer review: Decision Letter 0

**DOI:** 10.1371/journal.pgph.0007564.r001

**Authors:** Ruwandi Kariyawasam

23 Jun 2026

PGPH-D-26-01680

Interlaboratory comparison of a multiplex bead assay against the Chlamydia trachomatis antigen Pgp3 using two monoclonal antibody clones

PLOS Global Public Health

Dear Dr. Diana L. Martin,

Thank you for submitting your manuscript to PLOS Global Public Health. After careful consideration, we feel that it has merit but does not fully meet PLOS Global Public Health’s publication criteria as it currently stands - minor revisions required. Therefore, we invite you to submit a revised version of the manuscript that addresses the points raised during the review process.

==============================

The authors have done an excellent job at evaluating a novel multiplex bead assay using two monoclonal antibody clones targeting the C. trachomatis antigen, Pgp3. Very minor revisions required as indicated by the reviewers below, in addition to:

1) Rename Figure 2 (Line 172) as Supplementary Figure 1 and create panels A & B to indicate which antibody clone is evaluated (A: 1G1 B: 1C3).

2) Please ensure Table titles are listed above the Table and any footnotes, text etc. below the table

==============================

Please submit your revised manuscript by July 22, 2026. If you will need more time than this to complete your revisions, please reply to this message or contact the journal office at globalpubhealth@plos.org. When you're ready to submit your revision, log on to https://www.editorialmanager.com/pgph/ and select the 'Submissions Needing Revision' folder to locate your manuscript file.

Please include the following items when submitting your revised manuscript:

- A letter that responds to each point raised by the editor and reviewer(s). You should upload this letter as a separate file labeled 'Response to Reviewers'.
- A marked-up copy of your manuscript that highlights changes made to the original version. You should upload this as a separate file labeled 'Revised Manuscript with Track Changes'.
- An unmarked version of your revised paper without tracked changes. You should upload this as a separate file labeled 'Manuscript'.

Guidelines for resubmitting your figure files are available below the reviewer comments at the end of this letter.

As the corresponding author, your ORCID iD is verified in the submission system and will appear in the published article. PLOS supports the use of ORCID, and we encourage all coauthors to register for an ORCID iD and use it as well. Please encourage your coauthors to verify their ORCID iD within the submission system before final acceptance, as unverified ORCID iDs will not appear in the published article. *Only*  the individual author can complete the verification step; PLOS staff *cannot*  verify ORCID iDs on behalf of authors.

We look forward to receiving your revised manuscript.

Kind regards,

Dr. Ruwandi Kariyawasam

Academic Editor

PLOS Global Public Health

**Journal Requirements:**

1. Please amend your detailed Financial Disclosure statement. This is published with the article. It must therefore be completed in full sentences and contain the exact wording you wish to be published.

i. Please clarify all sources of financial support for your study. List the grants, grant numbers, and organizations that funded your study, including funding received from your institution. Please note that suppliers of material support, including research materials, should be recognized in the Acknowledgements section rather than in the Financial Disclosure.

ii. State the initials, alongside each funding source, of each author to receive each grant. For example: "This work was supported by the National Institutes of Health (####### to AM; ###### to CJ) and the National Science Foundation (###### to AM)."

iii. State what role the funders took in the study. If the funders had no role in your study, please state: “The funders had no role in study design, data collection and analysis, decision to publish, or preparation of the manuscript.”

iv. If any authors received a salary from any of your funders, please state which authors and which funders.

2. In the online submission form, you indicated that “The minimal data set [and accompanying code, where generated] is available from the corresponding author.”.

All PLOS journals now require all data underlying the findings described in their manuscript to be freely available to other researchers, either

1. In a public repository,

2. Within the manuscript itself, or

3. Uploaded as supplementary information.

This policy applies to all data except where public deposition would breach compliance with the protocol approved by your research ethics board. If your data cannot be made publicly available for ethical or legal reasons (e.g., public availability would compromise patient privacy), please explain your reasons by return email and your exemption request will be escalated to the editor for approval. Your exemption request will be handled independently and will not hold up the peer review process, but will need to be resolved should your manuscript be accepted for publication. One of the Editorial team will then be in touch if there are any issues.

3. We do not publish any copyright or trademark symbols that usually accompany proprietary names, eg (R), (C), or TM  (e.g. next to drug or reagent names). Please remove all instances of trademark/copyright symbols throughout the text, including Bio-Plex 200®, MAGPIX®, Intelliflex®, MagPlex®.

If the reviewer comments include a recommendation to cite specific previously published works, please review and evaluate these publications to determine whether they are relevant and should be cited. There is no requirement to cite these works unless the editor has indicated otherwise.

Please review your reference list to ensure that it is complete and correct. If you have cited papers that have been retracted, please include the rationale for doing so in the manuscript text, or remove these references and replace them with relevant current references. Any changes to the reference list should be mentioned in the rebuttal letter that accompanies your revised manuscript. If you need to cite a retracted article, indicate the article’s retracted status in the References list and also include a citation and full reference for the retraction notice.

**Additional Editor Comments (if provided):**

[Note: HTML markup is below. Please do not edit.]

Reviewers' comments:

Reviewer's Responses to Questions

**Comments to the Author**

1. Does this manuscript meet PLOS Global Public Health’s publication criteria?

Reviewer #1: Yes

Reviewer #2: Yes

**********

2. Has the statistical analysis been performed appropriately and rigorously?

Reviewer #1: Yes

Reviewer #2: Yes

**********

3. Have the authors made all data underlying the findings in their manuscript fully available (please refer to the Data Availability Statement at the start of the manuscript PDF file)?

The PLOS Data policy

Reviewer #1: Yes

Reviewer #2: Yes

**********

4. Is the manuscript presented in an intelligible fashion and written in standard English?

Reviewer #1: Yes

Reviewer #2: Yes

**********

**Reviewer #1:**  Minor revisions:

Line 122:

The percent change is noted as (final MFI – original MFI / original MFI). The way that this is currently written indicates that the original MFI / original MFI would result in 1 and then this would be subtracted from the final MFI. Likely it should be re-written as ([final MFI – original MFI] / original MFI) x 100.

Line 131 (and through-out):

Inter-lab precision may be better classified as reproducibility as per the ICH-Q2.

Figure 1:

On the y-axis, please add the temperature of sample storage to each of the different MFI-bg’s calculated for clarity.

Review notes as per reviewer guide:

What are the main claims of the paper and how significant are they for the discipline?

The manuscript demonstrates that Pgp3 mAb can be used to standardize Pgp3 antibody results between labs utilizing different multiplex bead assay (MBA) protocols and instrumentation. Two different Pgp3 mAb were also evaluated:1C3 and 1G1. After analysis, clone 1C3 yields more accurate and reproducible results and is more stable than clone 1G1.

Are the claims properly placed in the context of the previous literature? Have the authors treated the literature fairly?

Yes and yes. The authors have cited and discussed relevant published articles.

Do the data and analyses fully support the claims? If not, what other evidence is required?

Yes.

If a protocol is provided, for example for a randomized controlled trial, are there any important deviations from it? If so, have the authors explained adequately why the deviations occurred?

The authors have openly described the limitations of this study with detail.

PLOS Global Public Health encourages authors to publish detailed protocols and algorithms as supporting information online. Do any particular methods used in the manuscript warrant such treatment?

No.

If the paper is considered unsuitable for publication in its present form, does the study itself show sufficient potential that the authors should be encouraged to resubmit a revised version?

Yes, but I think in its present form warrants publications with a few minor changes.

Are original data deposited in appropriate repositories and accession/version numbers provided for genes, proteins, mutants, diseases, etc.?

N/A

Does the study conform to any relevant guidelines such as CONSORT, MIAME, QUORUM, STROBE, and the Fort Lauderdale agreement?

STARD does not apply as this is not a study of diagnostic accuracy as there were no data comparing sensitivity, specificity, predictive values nor AUC.

Are details of the methodology sufficient to allow the experiments to be reproduced?

An R package (coin package) was used for the statistical analysis of differences in absolute error between clones with the Wilcoxon-Mann-Whitney test. As per the reviewers guide a persistent id for any code that is used needs to be provided.

Is any software created by the authors freely available?

N/A

Is the manuscript well organized and written clearly enough to be accessible to non-specialists?

Yes.

**Reviewer #2:**  This study evaluates the interlaboratory performance of Pgp3 luminex-based multiplex bead serological assays using two chimeric anti-Pgp3 monoclonal antibodies (1G1 and 1C3) as standardized reference reagents, across different laboratories using independently developed protocols, with different operators and instrument types. The authors suggest that Pgp3 multiplex bead assays are highly robust for comparing different instruments and assay protocols, and the 1C3 clone may be the more reliable standard reagent that yields more accurate and reproducible results.

The study addresses a clinically relevant topic, an important need in Chlamydia trachomatis serology testing and connects its use in practical applications. The study is generally well designed. A notable strength of the study is the evaluation across multiple laboratories, operators, and instrument platforms using each laboratory's routine protocol. This approach provides an assessment of real-world performance and generalizability beyond a single-site setting which strengthens the evidence supporting the reagent's robustness.

Minor comments

The methodology for the stability assessment requires further clarification. The authors state designated specimens were transferred to a -20°C freezer at days 3, 7, and 21. It is not entirely clear if all concentrations of the Pgp3 mAb clones were included in the 'designated specimens' and how it was determined which concentrations were selected for storage at the different time points. Additional methodological detail would improve reproducibility and facilitate interpretation of the stability findings.

Figure 1 legend describes the stability of the clones at different storage conditions and timepoints; consider more explicit identification of clone type and post-storage timepoints in the figure panel labels to improve readability and reduce the need for cross-referencing the legend.

The abstract notes the 1C3 clone had 11/21 conditions within the desired accuracy range and the 1G1 had 18/21 conditions, however in the results, these conditions were reported as "out of range" (10/21 vs 3/21). Later on in the discussion section, these conditions were reported as "within range". The numbers are internally consistent but flipping the framing may be confusing for the reader. Consider maintaining consistent accuracy framing for clarity.

The paper describes in detail the different laboratory-specific protocols, with Table 2 highlighting the key technical differences. Could these differences explain the inter-lab variation noted in the raw data MFI readouts? Consider discussing whether specific protocol differences contributed to the variability. This would provide additional context for interpreting the results and further strengthen the robustness assessment.

Accuracy and precision readings outside of the desired range for the 1C3 clone were reported for the high concentration samples, but not explained. Could this be a limitation of the IC3 reagent? If not, providing a hypothesis of why this observed inaccuracy/imprecision occurred would reassure the readers it is not a limitation of the monoclonal antibody reagent.

Overall, the manuscript is well written and easy to follow, the methodology is generally rigorous, the data is presented clearly, and the conclusions are largely supported by the data. The comments made primarily relate to clarification and expansion of the discussion rather than concerns regarding study design or data interpretation.

**********

what does this mean?). If published, this will include your full peer review and any attached files.

**Do you want your identity to be public for this peer review?**  If you choose “no”, your identity will remain anonymous but your review may still be made public.

For information about this choice, including consent withdrawal, please see our Privacy Policy

Reviewer #1: **Yes:**  Ellen M. E. Sykes

Reviewer #2: **Yes:**  Ima Anugom

**********

[NOTE: If reviewer comments were submitted as an attachment file, they will be attached to this email and accessible via the submission site. Please log into your account, locate the manuscript record, and check for the action link "View Attachments". If this link does not appear, there are no attachment files.]

**Figure Resubmissions:**

# Author comment: Author response to Decision Letter 1

**DOI:** 10.1371/journal.pgph.0007564.r002

21 Jul 2026

Attachment Submitted filename: PGPH-D-26-01680 Response to Reviewers.docx

# Peer review: Decision Letter 1

**DOI:** 10.1371/journal.pgph.0007564.r003

**Authors:** Sarah Jose

10 Sep 2026

PGPH-D-26-01680R1

Interlaboratory comparison of a multiplex bead assay against the Chlamydia trachomatis antigen Pgp3 using two monoclonal antibody clones

PLOS Global Public Health

Dear Dr. Martin,

Thank you for submitting your manuscript to PLOS Global Public Health. After careful consideration, we feel that it has merit but does not fully meet PLOS Global Public Health’s publication criteria as it currently stands. Therefore, we invite you to submit a revised version of the manuscript that addresses the points raised during the review process.

Please find some minor suggestions from the reviewers below. Please revise your manuscript to address these comments, providing a point-by-point response to the reviewers upon resubmission.

Please submit your revised manuscript by Oct 09 2026 11:59PM. If you will need more time than this to complete your revisions, please reply to this message or contact the journal office at globalpubhealth@plos.org. When you're ready to submit your revision, log on to https://www.editorialmanager.com/pgph/ and select the 'Submissions Needing Revision' folder to locate your manuscript file.

Please include the following items when submitting your revised manuscript:

- A letter that responds to each point raised by the editor and reviewer(s). You should upload this letter as a separate file labeled 'Response to Reviewers'.
- A marked-up copy of your manuscript that highlights changes made to the original version. You should upload this as a separate file labeled 'Revised Manuscript with Track Changes'.
- An unmarked version of your revised paper without tracked changes. You should upload this as a separate file labeled 'Manuscript'.

Guidelines for resubmitting your figure files are available below the reviewer comments at the end of this letter.

As the corresponding author, your ORCID iD is verified in the submission system and will appear in the published article. PLOS supports the use of ORCID, and we encourage all coauthors to register for an ORCID iD and use it as well. Please encourage your coauthors to verify their ORCID iD within the submission system before final acceptance, as unverified ORCID iDs will not appear in the published article. *Only*  the individual author can complete the verification step; PLOS staff *cannot*  verify ORCID iDs on behalf of authors.

We look forward to receiving your revised manuscript.

Kind regards,

Sarah Jose, Ph.D.

Staff Editor

PLOS Global Public Health

**Journal Requirements:**

If the reviewer comments include a recommendation to cite specific previously published works, please review and evaluate these publications to determine whether they are relevant and should be cited. There is no requirement to cite these works unless the editor has indicated otherwise.

Please review your reference list to ensure that it is complete and correct. If you have cited papers that have been retracted, please include the rationale for doing so in the manuscript text, or remove these references and replace them with relevant current references. Any changes to the reference list should be mentioned in the rebuttal letter that accompanies your revised manuscript. If you need to cite a retracted article, indicate the article’s retracted status in the References list and also include a citation and full reference for the retraction notice.

**Additional Editor Comments (if provided):**

[Note: HTML markup is below. Please do not edit.]

Reviewers' comments:

Reviewer's Responses to Questions

**Comments to the Author**

Reviewer #1: (No Response)

Reviewer #2: All comments have been addressed

**********

publication criteria?

Reviewer #1: Yes

Reviewer #2: Yes

**********

3. Has the statistical analysis been performed appropriately and rigorously?

Reviewer #1: Yes

Reviewer #2: Yes

**********

4. Have the authors made all data underlying the findings in their manuscript fully available (please refer to the Data Availability Statement at the start of the manuscript PDF file)?

The PLOS Data policy

Reviewer #1: Yes

Reviewer #2: Yes

**********

5. Is the manuscript presented in an intelligible fashion and written in standard English?

Reviewer #1: Yes

Reviewer #2: Yes

**********

**Reviewer #1:**  Line 122:

How was the correlation coefficient calculated? Please note this information here.

Line 166:

The correlation coefficient has already been noted for each standard curve in Figure 1 for each clone as 0.999, etc. This statement in the Figure 1 legend is now unclear. Do the authors mean the residuals? Please revise.

**Reviewer #2:**  Please review for minor grammatical errors (e.g. lines 126 and 174).

**********

what does this mean?). If published, this will include your full peer review and any attached files.

**Do you want your identity to be public for this peer review?**  If you choose “no”, your identity will remain anonymous but your review may still be made public.

For information about this choice, including consent withdrawal, please see our Privacy Policy

Reviewer #1: **Yes:**  Ellen M. E. Sykes

Reviewer #2: **Yes:**  Ima Anugom

**********

[NOTE: If reviewer comments were submitted as an attachment file, they will be attached to this email and accessible via the submission site. Please log into your account, locate the manuscript record, and check for the action link "View Attachments". If this link does not appear, there are no attachment files.]

**Figure Resubmissions:**

# Author comment: Author response to Decision Letter 2

**DOI:** 10.1371/journal.pgph.0007564.r004

16 Sep 2026

Attachment Submitted filename: PLOS GH Response to Reviewers 09162026.docx

# Peer review: Decision Letter 2

**DOI:** 10.1371/journal.pgph.0007564.r005

**Authors:** Mara Jana Broadhurst

15 Oct 2026

Interlaboratory comparison of a multiplex bead assay against the Chlamydia trachomatis antigen Pgp3 using two monoclonal antibody clones

PGPH-D-26-01680R2

Dear Dr. Martin,

We are pleased to inform you that your manuscript 'Interlaboratory comparison of a multiplex bead assay against the Chlamydia trachomatis antigen Pgp3 using two monoclonal antibody clones' has been provisionally accepted for publication in PLOS Global Public Health.

Before your manuscript can be formally accepted you will need to complete some formatting changes, which you will receive in a follow up email. A member of our team will be in touch with a set of requests.

Please note that your manuscript will not be scheduled for publication until you have made the required changes, so a swift response is appreciated.

IMPORTANT: The editorial review process is now complete. PLOS will only permit corrections to spelling, formatting or significant scientific errors from this point onwards. Requests for major changes, or any which affect the scientific understanding of your work, will cause delays to the publication date of your manuscript.

If your institution or institutions have a press office, please notify them about your upcoming paper to help maximize its impact. If they'll be preparing press materials, please inform our press team as soon as possible -- no later than 48 hours after receiving the formal acceptance. Your manuscript will remain under strict press embargo until 2 pm Eastern Time on the date of publication. For more information, please contact globalpubhealth@plos.org.

Thank you again for supporting Open Access publishing; we are looking forward to publishing your work in PLOS Global Public Health.

Best regards,

Mara Jana Broadhurst, M.D., Ph.D.

Academic Editor

PLOS Global Public Health

***********************************************************

Reviewer Comments (if any, and for reference):

Reviewer's Responses to Questions

**Comments to the Author**

Reviewer #1: All comments have been addressed

Reviewer #2: (No Response)

**********

publication criteria?

Reviewer #1: Yes

Reviewer #2: Yes

**********

3. Has the statistical analysis been performed appropriately and rigorously?

Reviewer #1: Yes

Reviewer #2: Yes

**********

4. Have the authors made all data underlying the findings in their manuscript fully available (please refer to the Data Availability Statement at the start of the manuscript PDF file)?

The PLOS Data policy

Reviewer #1: Yes

Reviewer #2: Yes

**********

5. Is the manuscript presented in an intelligible fashion and written in standard English?

Reviewer #1: Yes

Reviewer #2: Yes

**********

Reviewer #1: Thanks for addressing all of our comments.

Reviewer #2: (No Response)

**********

what does this mean?). If published, this will include your full peer review and any attached files.

**Do you want your identity to be public for this peer review?**  If you choose “no”, your identity will remain anonymous but your review may still be made public.

For information about this choice, including consent withdrawal, please see our Privacy Policy

Reviewer #1: **Yes:**  Ellen M. E. Sykes

Reviewer #2: **Yes:**  Ima Anugom

**********
